# Comparative Cardiovascular Outcomes and Safety Profiles of Glucagon-Like Peptide-1 Receptor Agonists in Type 2 Diabetes Mellitus: A Systematic Review and Meta-Analysis of Randomized Trials

**DOI:** 10.7759/cureus.111723

**Published:** 2026-06-29

**Authors:** Samih Abdelmutalab Mohamed Abdalla, Hind Osman Ali Mohammed, Eltayeb Osman E Omer, Ibrahim Obied Ibrahim Ahmed, Wadah Ahmed Osman Ahmed, Khalid Omer Taha Ginawi, Riham Elgaali Mohamed Abdelfattah, Ahmed Abdalla Zarrouq Yousif, Khadija Ibrahim Ahmed Ismail

**Affiliations:** 1 Internal Medicine, Al Hammadi Hospital, Riyadh, SAU; 2 General Medicine, University Hospital Waterford, Waterford, IRL; 3 Endocrinology, Dr. Sulaiman Al Habib Hospital, Riyadh, SAU; 4 Internal Medicine, Suwair Hospital, Al-Jouf, SAU; 5 Internal Medicine, Kalba Hospital, Sharjah, ARE; 6 Internal Medicine, Najran Health Cluster, Ministry of Health, Najran, SAU; 7 General Practice, Taha Medical Centre, Abu Dhabi, ARE; 8 Endocrinology, Sligo University Hospital, Sligo, IRL; 9 Internal Medicine, Raqamat Specialist Hospital, Najran, SAU

**Keywords:** cardiovascular outcomes, glp-1 receptor agonists, major adverse cardiovascular events, systematic review, type 2 diabetes mellitus

## Abstract

Cardiovascular disease is the leading cause of morbidity and mortality in type 2 diabetes mellitus (T2DM). Glucagon-like peptide-1 receptor agonists (GLP-1 RAs) have been assessed in several large cardiovascular outcome trials (CVOTs); however, the individual agents differ in molecular structure, pharmacology, and the populations studied, and their reported effects range from neutral to clearly beneficial. This review compares the cardiovascular efficacy and safety of GLP-1 RAs across all eligible randomized, placebo-controlled CVOTs in T2DM and quantitatively pools the primary outcome.

Following the PRISMA 2020 statement, ClinicalTrials.gov, MEDLINE, Embase, and the Cochrane CENTRAL were searched from inception to 15 December 2025 for randomized, double-blind, placebo-controlled CVOTs comparing a GLP-1 RA with placebo in adults with T2DM and reporting adjudicated major adverse cardiovascular events (MACE) as a primary or co-primary endpoint. The protocol was not prospectively registered. Two reviewers independently performed screening, data extraction, and risk-of-bias assessment (Cochrane RoB 2), and certainty of evidence was rated using the Grading of Recommendations Assessment, Development, and Evaluation (GRADE). Findings were synthesized narratively and, for the primary MACE outcome, pooled using a DerSimonian-Laird random-effects model on the log-hazard-ratio scale, with heterogeneity quantified by I² and Cochran's Q, leave-one-out sensitivity analysis, and small-study assessment by funnel plot and Egger's test.

Nine trials enrolling 69,730 participants met the eligibility criteria. The primary MACE hazard ratio favored the GLP-1 RA in eight of nine trials (range = 0.73-1.02) and reached statistical superiority in five. Random-effects meta-analysis yielded a pooled MACE hazard ratio of 0.86 (95% CI: 0.82-0.92; P < 0.001), corresponding to a 14% relative risk reduction, with moderate heterogeneity (I² = 37%; Cochran's Q = 12.7, P = 0.12) and a 95% prediction interval of 0.75-1.00. The pooled estimate was robust to leave-one-out analysis (0.85-0.88), and no small-study asymmetry was detected (Egger's P = 0.13). The composite benefit was driven by reductions in myocardial infarction and stroke, with a neutral effect on hospitalization for heart failure. Gastrointestinal adverse events were the most common class effect; a class-level excess of gallbladder and biliary disease was also evident, and a diabetic retinopathy signal was observed with subcutaneous semaglutide. Eight trials were judged to be at low risk of bias, and the certainty of evidence was moderate for the principal cardiovascular outcomes.

In adults with T2DM, GLP-1 RAs as a class reduce the risk of MACE by approximately 14% with an acceptable safety profile, supporting their role as a cornerstone of cardiovascular risk reduction. Between-agent and between-trial heterogeneity preclude a definitive ranking of individual agents.

## Introduction and background

Type 2 diabetes mellitus (T2DM) is among the strongest modifiable contributors to atherosclerotic cardiovascular disease, conferring roughly a two-fold excess risk of coronary heart disease, ischemic stroke, and vascular death that is only partly explained by conventional risk factors [1]. For decades, the central therapeutic assumption was that improving glycemic control would translate directly into fewer macrovascular events. That assumption proved only partially correct: long-term follow-up of intensive glucose-lowering strategies demonstrated durable reductions in microvascular complications but a comparatively modest and delayed effect on myocardial infarction and cardiovascular mortality [2]. This dissociation between glycemic and cardiovascular benefit reframed the problem, shifting attention away from glucose-lowering as a surrogate and toward the cardiovascular effects of specific drug classes assessed against hard clinical endpoints.

The regulatory response to this shift was decisive. Following the 2008 United States Food and Drug Administration guidance requiring that all new glucose-lowering agents demonstrate cardiovascular safety in dedicated outcome trials, a generation of large, event-driven, placebo-controlled cardiovascular outcome trials (CVOTs, randomized trials powered for adjudicated cardiovascular endpoints rather than glycemic surrogates) was launched [3]. Throughout this review, major adverse cardiovascular events (MACE) denote the adjudicated composite of cardiovascular death, nonfatal myocardial infarction, and nonfatal stroke (three-point MACE), with four-point definitions additionally including hospitalization for unstable angina. Glucagon-like peptide-1 receptor agonists (GLP-1 RAs) emerged as a particularly compelling class within this framework. Beyond their incretin-mediated, glucose-dependent stimulation of insulin secretion and suppression of glucagon, GLP-1 RAs exert pleiotropic actions-weight reduction, lowering of systolic blood pressure, attenuation of vascular inflammation, improvement of endothelial function, and favorable effects on the atherosclerotic plaque, that provide a coherent biological rationale for cardiovascular protection independent of glycemia [4]. More recent mechanistic syntheses attribute these vascular effects to glucagon-like peptide-1 (GLP-1)-receptor-dependent anti-inflammatory signaling in endothelial cells, monocytes, and macrophages, including reduced expression of adhesion molecules and suppression of pro-atherogenic cytokine pathways [5]. These mechanistic expectations required confirmation in adequately powered randomized trials with adjudicated cardiovascular endpoints.

The class comprises structurally distinct agents that differ in their backbone and pharmacokinetics: human GLP-1 analogues (liraglutide, semaglutide (subcutaneous and oral), dulaglutide, and albiglutide) and exendin-4-based compounds (lixisenatide, exenatide, and efpeglenatide), administered by daily or weekly subcutaneous injection or, for semaglutide, orally. These differences are clinically relevant because they appear to track, imperfectly, with the magnitude of cardiovascular benefit observed across trials. The CVOT program for this class began cautiously. The first completed trial established cardiovascular safety but not benefit for lixisenatide [6], whereas subsequent trials of liraglutide and subcutaneous semaglutide reported significant reductions in MACE, shifting the clinical narrative from safety to efficacy [7,8]. The accumulated evidence has since reshaped clinical practice guidelines, which now recommend GLP-1 RAs with proven cardiovascular benefit for patients with T2DM and established or high risk of atherosclerotic disease [9], a position reaffirmed in the most contemporary guidance, including the American Diabetes Association Standards of Care in Diabetes 2025 [10] and the 2023 European Society of Cardiology guidelines for the management of cardiovascular disease in patients with diabetes [11], and reinforced by class-level meta-analyses suggesting a consistent reduction in cardiovascular and renal events [12].

Nevertheless, the individual agents differ in molecular structure, pharmacokinetics, route and frequency of administration, and the cardiovascular risk profile of the populations studied, and the trial results themselves range from frank neutrality to substantial benefit. A structured synthesis of the randomized evidence is therefore warranted. This systematic review and meta-analysis compiles and compares the cardiovascular efficacy and safety of GLP-1 RAs across all eligible randomized CVOTs in T2DM. Its distinct contribution relative to prior syntheses is two-fold: it incorporates the most recently reported large CVOT that was not available to earlier pooled analyses, and it complements the narrative comparison of agents and populations with a contemporary random-effects meta-analysis, formal heterogeneity quantification, sensitivity analyses, and a summary of findings table, thereby updating and extending the existing evidence base.

## Review

Methods

Study Design

This systematic review and meta-analysis was designed, conducted, and reported in accordance with the Preferred Reporting Items for Systematic Reviews and Meta-Analyses (PRISMA) 2020 statement [13]. A review protocol specifying the research question, eligibility criteria, search strategy, and outcomes of interest was developed a priori. The protocol was not prospectively registered in the International Prospective Register of Systematic Reviews (PROSPERO) or the Open Science Framework; it is acknowledged as a limitation.

Eligibility Criteria (PICOS Framework)

Eligibility was defined using the Population, Intervention, Comparator, Outcomes, and Study design (PICOS) framework [14], summarized in Table 1.

**Table 1:** Eligibility criteria according to the PICOS framework. PICOS: Population, Intervention, Comparator, Outcomes, and Study design; GLP-1: glucagon-like peptide-1; GIP: gastric inhibitory polypeptide; MACE: major adverse cardiovascular events; T2DM: type 2 diabetes mellitus.

Domain	Inclusion criteria	Exclusion criteria
Population	Adults (≥18 years) with type 2 diabetes mellitus	Type 1 diabetes; populations without diabetes; mixed cohorts where T2DM data are not separable
Intervention	A GLP-1 receptor agonist added to standard care	Non-GLP-1 agents; dual GIP/GLP-1 co-agonists; GLP-1 receptor agonist used solely for weight management without T2DM
Comparator	Placebo plus standard care	Active glucose-lowering comparator only (no placebo arm)
Outcomes	Adjudicated MACE (3- or 4-point) as a primary or co-primary endpoint, with cardiovascular and safety outcomes reported	Studies reporting only surrogate or biochemical endpoints; primary renal-only endpoint trials
Study design	Randomized, double-blind, placebo-controlled cardiovascular outcome trials (full peer-reviewed publications)	Reviews, meta-analyses, observational studies, post hoc/secondary analyses, conference abstracts, preprints, protocols

Information Sources and Search Strategy

A systematic search of ClinicalTrials.gov, MEDLINE (via PubMed), Embase, and the Cochrane Central Register of Controlled Trials (CENTRAL) was conducted from database inception to 15 December 2025, which was the date of the final search. The search combined controlled vocabulary and free-text terms structured around two concept blocks: (i) the drug class and individual agents, and (ii) the cardiovascular outcome and randomized design. No language restriction was applied; results were limited to human studies. The complete, database-specific electronic search strategies, including Boolean operators, MeSH/Emtree terms, field tags, filters, and limits, are reported in full in a supplementary table included in the Appendices.

Study Selection

**Figure 1:**
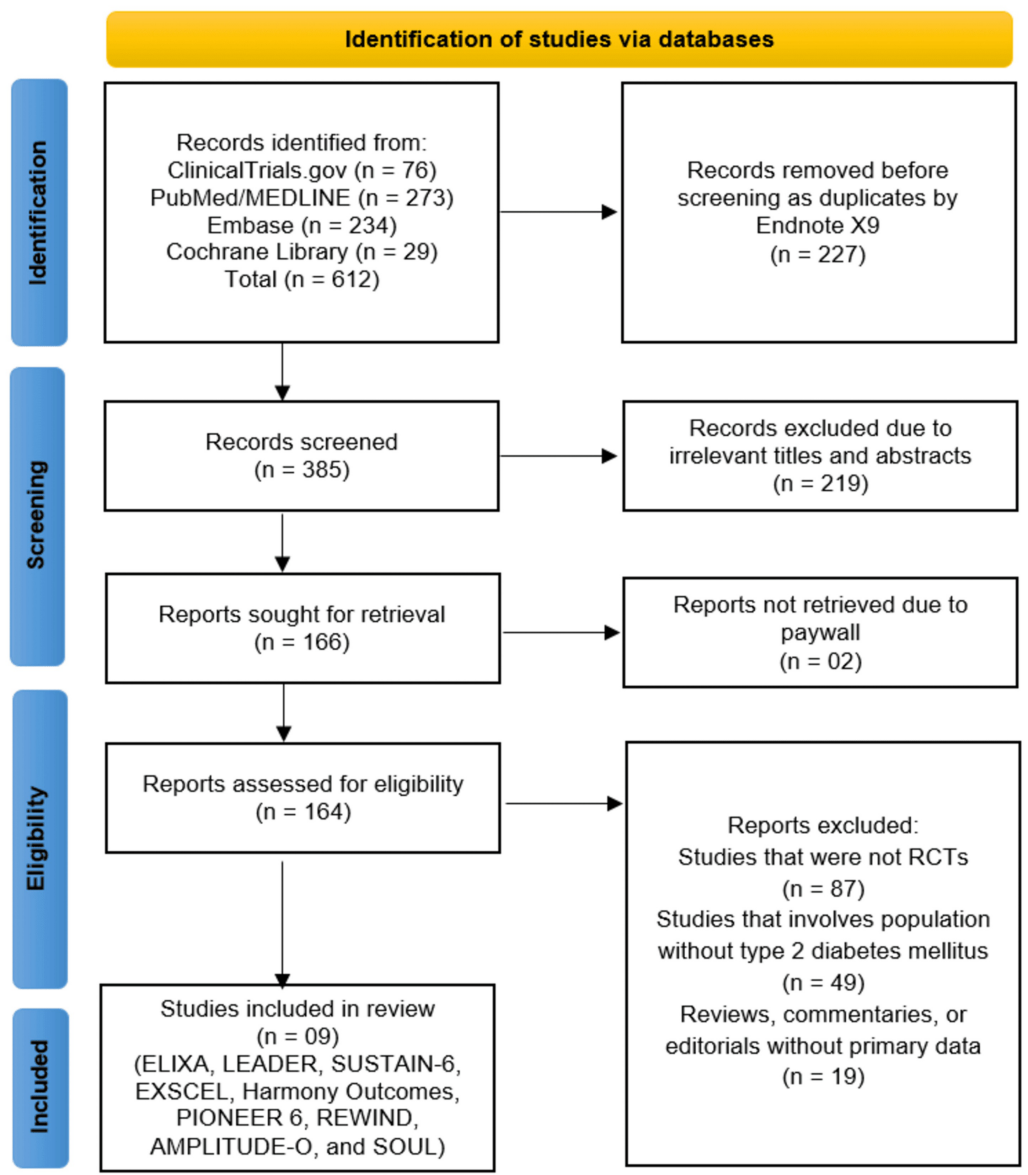
PRISMA 2020 flow diagram of study identification, screening, and inclusion.

Records retrieved from all sources were de-duplicated and screened in two stages by two independent reviewers. Titles and abstracts were screened first, after which full texts of potentially eligible records were assessed against the eligibility criteria. Inter-reviewer agreement at full-text screening was high (Cohen's κ = 0.89). Disagreements at either stage were resolved by discussion and, where necessary, adjudication by a third reviewer. Two reports could not be retrieved owing to paywall restrictions; both were conference-proceedings records whose abstracts described secondary or pooled analyses of trials already captured by the search rather than independent primary CVOTs, and, because all nine landmark GLP-1 RA CVOTs were identified and included, their unavailability is unlikely to have materially influenced the review findings (also discussed under Limitations). The selection process and the corresponding record counts at each stage are reported in the PRISMA 2020 flow diagram (Figure 1).

Data Extraction

A standardized data-extraction form was piloted independently by the two reviewers on a random sample of three included trials and refined for clarity and completeness before full extraction; agreement after piloting was satisfactory, and the form was considered calibrated. Using the piloted form, the following were captured in duplicate from each included trial: trial name, registration number, year and journal of publication, intervention and dose, comparator, sample size, key baseline characteristics (age, sex, diabetes duration, baseline glycosylated hemoglobin (HbA1c), proportion with established cardiovascular disease), median follow-up, primary endpoint definition, the hazard ratio (HR) and 95% confidence interval (CI) for the primary MACE outcome, individual MACE components, all-cause mortality, and prespecified safety outcomes. Discrepancies were reconciled against the primary publication.

Risk-of-Bias Assessment

The methodological quality of each included trial was independently appraised by two reviewers using the revised Cochrane risk-of-bias tool for randomized trials (RoB 2) [15] across five domains, i.e., (D1) randomization process, (D2) deviations from intended interventions, (D3) missing outcome data, (D4) measurement of the outcome, and (D5) selection of the reported result, with a domain-level and overall judgement of low risk, some concerns, or high risk. Disagreements were resolved by consensus.

Certainty of Evidence (GRADE)

The certainty of evidence for each outcome was rated using the Grading of Recommendations Assessment, Development, and Evaluation (GRADE) approach [16], across risk of bias, inconsistency, indirectness, imprecision, and publication bias. As all included studies were randomized trials, the body of evidence for each outcome began at high certainty and was rated down where serious limitations were identified. A GRADE summary of findings table with anticipated absolute effects is provided.

Data Synthesis

Results were synthesized narratively and presented in structured summary tables, with treatment effects reported as HRs with 95% CIs as published in the primary trials. In addition, and in response to the comparability of the included CVOTs, a quantitative synthesis of the primary MACE outcome was performed. Trial-level log-hazard ratios and their standard errors (derived from the published 95% CIs) were pooled using the generic inverse-variance method under a DerSimonian-Laird random-effects model, chosen a priori in anticipation of clinical and methodological heterogeneity across agents and populations. Statistical heterogeneity was quantified using Cochran's Q (with its P-value), the I² statistic, and the between-study variance τ²; a 95% prediction interval was calculated to express the expected dispersion of true effects. The robustness of the pooled estimate was examined by leave-one-out sensitivity analysis. Small-study effects and potential publication bias were assessed visually by a contour-style funnel plot and formally by Egger's regression test, acknowledging the limited power of these methods with fewer than 10 studies. A fixed-effect estimate was computed as a sensitivity analysis. Anticipated absolute effects (events per 1000, absolute risk reduction, and number needed to treat) were derived by applying the pooled relative effect to a representative assumed control-group risk for the GRADE summary of findings table. Two-sided P < 0.05 was considered statistically significant. Because the included trials differ substantially in agent pharmacology, population risk, and endpoint definition, the pooled estimate is interpreted as a class-level average effect and is presented alongside, rather than in place of, the narrative comparison of individual agents. Analyses were conducted in Python (NumPy/SciPy) (Python Software Foundation, Wilmington, DE).

Results

Study Selection and Characteristics

The database search identified 612 records, including 273 from PubMed/MEDLINE, 234 from Embase, 76 from ClinicalTrials.gov, and 29 from the Cochrane Library, of which 227 duplicates were removed using EndNote X9, leaving 385 records for title and abstract screening. Screening excluded 219 records as irrelevant, and 166 reports were sought for retrieval, of which two could not be obtained because of paywall restrictions (both conference proceeding records were judged ineligible on the basis of their abstracts; see Study selection). The remaining 164 full-text reports were assessed for eligibility, and 155 were excluded: 87 were not randomized controlled trials, 49 enrolled populations without T2DM, and 19 were reviews, commentaries, or editorials lacking primary data. Nine randomized, double-blind, placebo-controlled CVOTs met all eligibility criteria and were included: ELIXA [6], LEADER [7], SUSTAIN-6 [8], EXSCEL [17], Harmony Outcomes [18], PIONEER 6 [19], REWIND [20], AMPLITUDE-O [21], and SOUL [22]. Together, these trials randomized 69,730 participants with T2DM and were published between 2015 and 2025. The design and baseline characteristics of the included trials are presented in Table 2, and their primary and key secondary outcomes in Table 3.

**Table 2:** Design and baseline characteristics of the included trials. ACS: acute coronary syndrome; ASCVD: atherosclerotic cardiovascular disease; CKD: chronic kidney disease; CV: cardiovascular; CVD: cardiovascular disease; ER: extended release; s.c.: subcutaneous; T2DM: type 2 diabetes mellitus; mg: milligrams; CV/renal: cardiovascular and/or renal; CVD/CKD: cardiovascular disease and/or chronic kidney disease.

Trial (year, Ref.)	Agent (dose)	N	Population	Prior CVD	Median follow-up
ELIXA (2015) [6]	Lixisenatide (daily)	6,068	T2DM + recent ACS	100% (ACS)	~2.1 years
LEADER (2016) [7]	Liraglutide 1.8 mg (daily)	9,340	T2DM, high CV risk	~81%	3.8 years
SUSTAIN-6 (2016) [8]	Semaglutide s.c. 0.5/1.0 mg (weekly)	3,297	T2DM, high CV risk	~83% (CVD/CKD)	2.1 years
EXSCEL (2017) [17]	Exenatide ER 2 mg (weekly)	14,752	T2DM, broad CV risk	~73%	3.2 years
Harmony (2018) [18]	Albiglutide (weekly)	9,463	T2DM + established CVD	100%	1.6 years
PIONEER 6 (2019) [19]	Oral semaglutide 14 mg (daily)	3,183	T2DM, high CV risk	~85% (CVD/CKD)	1.3 years
REWIND (2019) [20]	Dulaglutide 1.5 mg (weekly)	9,901	T2DM, CVD, or risk factors	31.5%	5.4 years
AMPLITUDE-O (2021) [21]	Efpeglenatide 4/6 mg (weekly)	4,076	T2DM, high CV/renal risk	~90%	1.8 years
SOUL (2025) [22]	Oral semaglutide ≤14 mg (daily)	9,650	T2DM + ASCVD/CKD	High (ASCVD/CKD)	~4.1 years

**Table 3:** Primary MACE and key secondary cardiovascular outcomes. CI: confidence interval; CV: cardiovascular; HF: heart failure; HR: hazard ratio; MACE: major adverse cardiovascular events; MI: myocardial infarction. Hazard ratios are for the primary composite outcome as reported in each trial; ELIXA used a four-point MACE, additionally including hospitalization for unstable angina. “—” indicates not tabulated as a primary or confirmatory secondary outcome. Numbers in parentheses in the “Notable findings” column are hazard ratios with 95% confidence intervals (HR, 95% CI).

Trial (Ref.)	Primary endpoint	MACE HR (95% CI)	Result	All-cause mortality HR (95% CI)	Notable findings
ELIXA [6]	4-point MACE	1.02 (0.89-1.17)	Non-inferior; not superior	0.94 (0.78-1.13)	Neutral; no effect on HF hospitalization
LEADER [7]	3-point MACE	0.87 (0.78-0.97)	Superior	0.85 (0.74-0.97)	CV death reduced (0.78, 0.66-0.93)
SUSTAIN-6 [8]	3-point MACE	0.74 (0.58-0.95)	Non-inferior (superiority post hoc)	1.05 (0.74-1.50)	Stroke reduced; retinopathy ↑ (1.76, 1.11-2.78)
EXSCEL [17]	3-point MACE	0.91 (0.83-1.00)	Non-inferior; not superior (p=0.06)	0.86 (0.77-0.97)	All-cause mortality was nominally lower
Harmony [18]	3-point MACE	0.78 (0.68-0.90)	Superior	0.95 (0.79-1.16)	Benefit driven by myocardial infarction
PIONEER 6 [19]	3-point MACE	0.79 (0.57-1.11)	Non-inferior	0.51 (0.31-0.84)	CV and all-cause death were lower
REWIND [20]	3-point MACE	0.88 (0.79-0.99)	Superior	0.90 (0.80-1.01)	Benefit driven by stroke; lowest baseline CVD
AMPLITUDE-O [21]	3-point MACE	0.73 (0.58-0.92)	Superior	—	Renal composite reduced (0.68, 0.57-0.79)
SOUL [22]	3-point MACE	0.86 (0.77-0.96)	Superior	—	Benefit driven by nonfatal MI

Cardiovascular Efficacy

Across the nine trials, the point estimates for the primary MACE outcome favored GLP-1 RA therapy in eight of nine trials, with hazard ratios ranging from 0.73 to 1.02. The single neutral trial, ELIXA, evaluated lixisenatide after recent acute coronary syndrome and demonstrated non-inferiority without cardiovascular benefit (HR: 1.02, 95% CI: 0.89-1.17) [6]. Statistically significant superiority over placebo was established in five trials: LEADER (HR: 0.87, 95% CI: 0.78-0.97) [7], Harmony Outcomes (HR: 0.78, 95% CI: 0.68-0.90) [18], REWIND (HR: 0.88, 95% CI: 0.79-0.99) [20], AMPLITUDE-O (HR: 0.73, 95% CI: 0.58-0.92) [21], and SOUL (HR: 0.86, 95% CI: 0.77-0.96) [22]. SUSTAIN-6 reported a 26% relative reduction in MACE (HR: 0.74, 95% CI: 0.58-0.95) that met the prespecified non-inferiority margin, with superiority shown only post hoc [8]. EXSCEL (HR: 0.91, 95% CI: 0.83-1.00) [17] and PIONEER 6 (HR: 0.79, 95% CI: 0.57-1.11) [19] confirmed safety with point estimates favoring the active agent but did not reach superiority; in EXSCEL, the comparison narrowly missed significance (p = 0.06) (Table 3).

A notable feature of the dataset is the divergence in the principal driver of the composite endpoint: benefit in Harmony Outcomes and SOUL was driven predominantly by myocardial infarction [18,22], whereas in REWIND, it was driven chiefly by stroke, consistent with the stroke reduction in SUSTAIN-6 [8,20]. This pattern suggests that the class effect is expressed through complementary rather than identical pathways across agents.

Quantitative Synthesis

**Figure 2:**
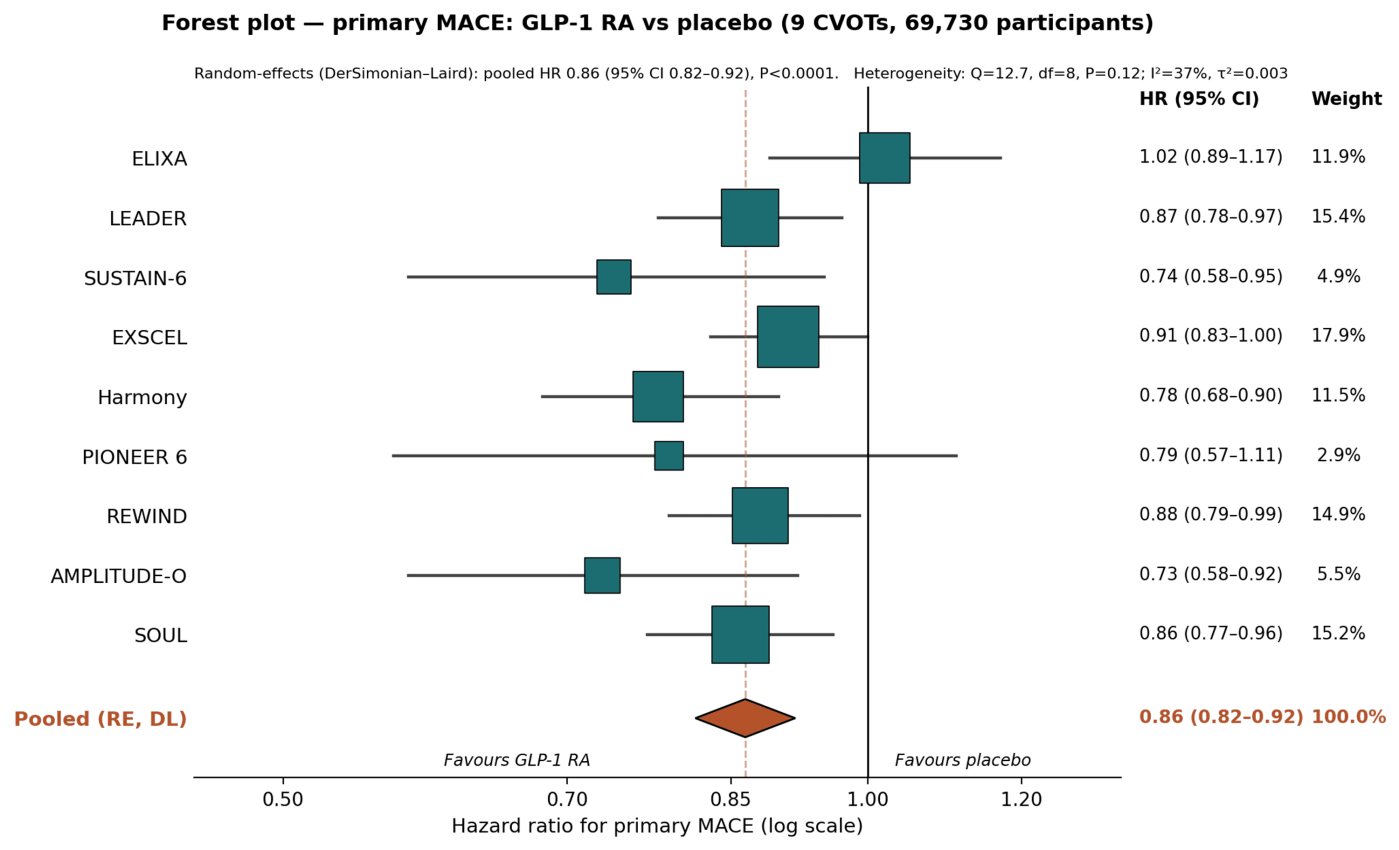
Random-effects forest plot of primary MACE (GLP-1 RA vs. placebo) across nine CVOTs. Marker size is proportional to study weight; the diamond represents the pooled DerSimonian–Laird estimate. ELIXA [6], LEADER [7], SUSTAIN-6 [8], EXSCEL [17], Harmony [18], PIONEER 6 [19], REWIND [20], AMPLITUDE-O [21], and SOUL [22]. MACE: major adverse cardiovascular events; GLP-1 RA: glucagon-like peptide-1 receptor agonist; CVOTs: cardiovascular outcome trials.

**Figure 3:**
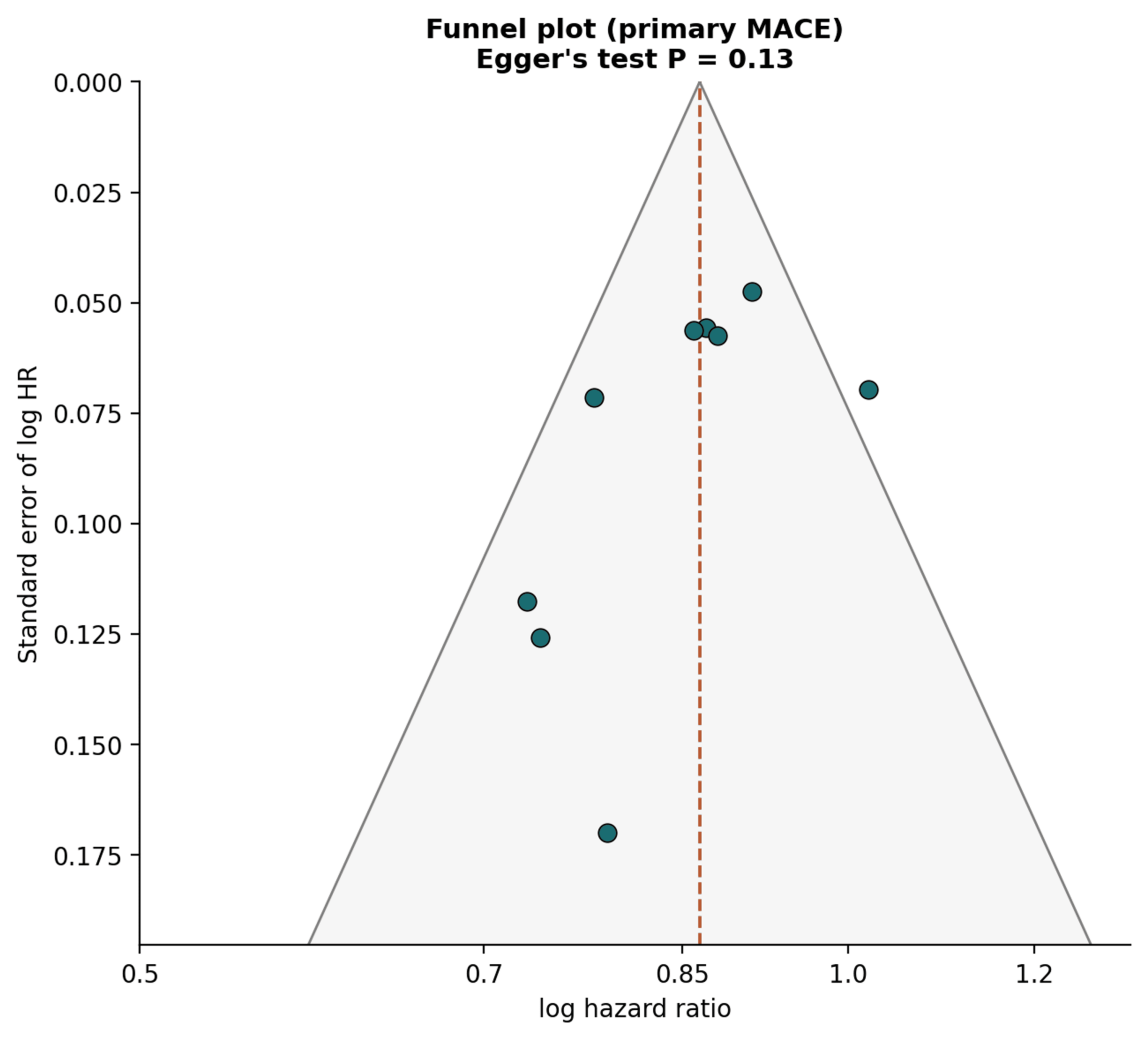
Funnel plot of the primary MACE log-hazard ratios against their standard errors. Egger's test P = 0.13, indicating no significant asymmetry. MACE: major adverse cardiovascular events.

Pooling the primary MACE hazard ratios from all nine trials under a DerSimonian-Laird random-effects model yielded a class-level hazard ratio of 0.86 (95% CI: 0.82-0.92; P < 0.001), corresponding to an approximately 14% relative reduction in MACE for GLP-1 RA therapy versus placebo (Figure 2). Heterogeneity was moderate and not statistically significant (Cochran's Q = 12.7, df = 8, P = 0.12; I² = 37.0%; τ² = 0.003), and the 95% prediction interval (0.75-1.00) indicates that, while the average effect is protective, the true effect in an individual future trial could range from a substantial benefit to a near-null result, reflecting genuine between-agent variation. The fixed-effect estimate was essentially identical (HR: 0.87, 95% CI: 0.83-0.91), and leave-one-out analysis confirmed robustness, with the pooled HR remaining between 0.85 and 0.88 and the 95% CI excluding unity regardless of which trial was omitted. The largest single contributors to the pooled estimate were EXSCEL (17.9%), LEADER (15.4%), SOUL (15.2%), and REWIND (14.9%). Visual inspection of the funnel plot (Figure 3) showed broadly symmetrical scatter, and Egger's regression test provided no evidence of small-study effects or publication bias (intercept: −1.90, P = 0.13), although the power of this assessment is limited by the small number of trials.

Mortality

Reductions in all-cause mortality were observed in several trials. LEADER demonstrated a significant reduction in death from any cause (HR: 0.85, 95% CI: 0.74-0.97) alongside a reduction in cardiovascular death (HR: 0.78, 95% CI: 0.66-0.93) [7]. EXSCEL reported a nominally significant reduction in all-cause mortality (HR: 0.86, 95% CI: 0.77-0.97) despite a neutral primary efficacy result [17], and PIONEER 6 showed lower cardiovascular and all-cause mortality (all-cause death, HR: 0.51, 95% CI: 0.31-0.84), albeit on a small number of events [19]. In the remaining trials, mortality estimates were directionally favorable but not statistically significant.

Population Heterogeneity

The proportion of participants with established cardiovascular disease varied substantially, from 31.5% in REWIND, the trial with the largest primary-prevention component and the longest follow-up (5.4 years), to 100% in ELIXA and Harmony Outcomes [6,18,20]. Despite a lower-risk, broader population, REWIND nonetheless demonstrated significant MACE reduction, indicating that the benefit is not confined to secondary-prevention populations [20]. Trials with shorter follow-up and fewer accrued events (PIONEER 6, EXSCEL) were correspondingly less likely to demonstrate statistical superiority even where point estimates favored therapy [17,19].

Safety

The included trials consistently established the cardiovascular safety of GLP-1 RAs, with no trial demonstrating an excess of MACE relative to placebo. Gastrointestinal adverse events, including nausea, vomiting, diarrhea, and constipation, were the most frequently reported class-related adverse effects and the leading cause of treatment discontinuation, most prominent with the oral formulation in PIONEER 6 and SOUL [19,22]. Rates of acute pancreatitis, pancreatic cancer, and medullary thyroid carcinoma did not differ significantly from placebo in any trial reporting these outcomes [6,17,18]. A trial-specific signal arose in SUSTAIN-6, in which subcutaneous semaglutide was associated with a significant increase in diabetic retinopathy complications (HR: 1.76, 95% CI: 1.11-2.78), attributed in part to the magnitude and rapidity of early glycemic reduction rather than to direct retinal toxicity [8]. Hospitalization for heart failure was neutral across trials reporting this endpoint, including ELIXA and EXSCEL [6,17].

Several additional adverse events of clinical relevance were considered. Gallbladder and biliary disease constitutes a recognized class effect: a large meta-analysis of 76 randomized trials reported a 37% relative increase in gallbladder or biliary disease with GLP-1 RAs (relative risk: 1.37), corresponding to a small absolute excess of roughly 27 additional cases per 10,000 person-years, with the highest risk at higher doses and longer durations [23]; consistent with this, LEADER reported a significant excess of acute gallbladder or biliary events with liraglutide [7]. Cholelithiasis and cholecystitis are the dominant components of this signal and are thought to relate to weight loss and altered biliary dynamics. Severe (level-2/3) hypoglycemia was uncommon across the CVOTs and not increased by GLP-1 RAs relative to placebo, consistent with their glucose-dependent mechanism, with events largely attributable to concomitant insulin or sulfonylurea use. Injection-site reactions were generally mild and most frequently reported with the exendin-based and weekly agents, including exenatide and efpeglenatide. Acute kidney injury was not consistently increased; where reported, it was most often linked to volume depletion in the context of gastrointestinal intolerance, while the renal composite was in fact reduced in AMPLITUDE-O (0.68, 0.57-0.79) [21]. Treatment discontinuation rates were driven principally by gastrointestinal intolerance and were numerically higher with oral semaglutide. Reporting of these adverse events was, however, inconsistent across the included trials, which constrains formal comparison (see Limitations).

Risk of Bias

**Figure 4:**
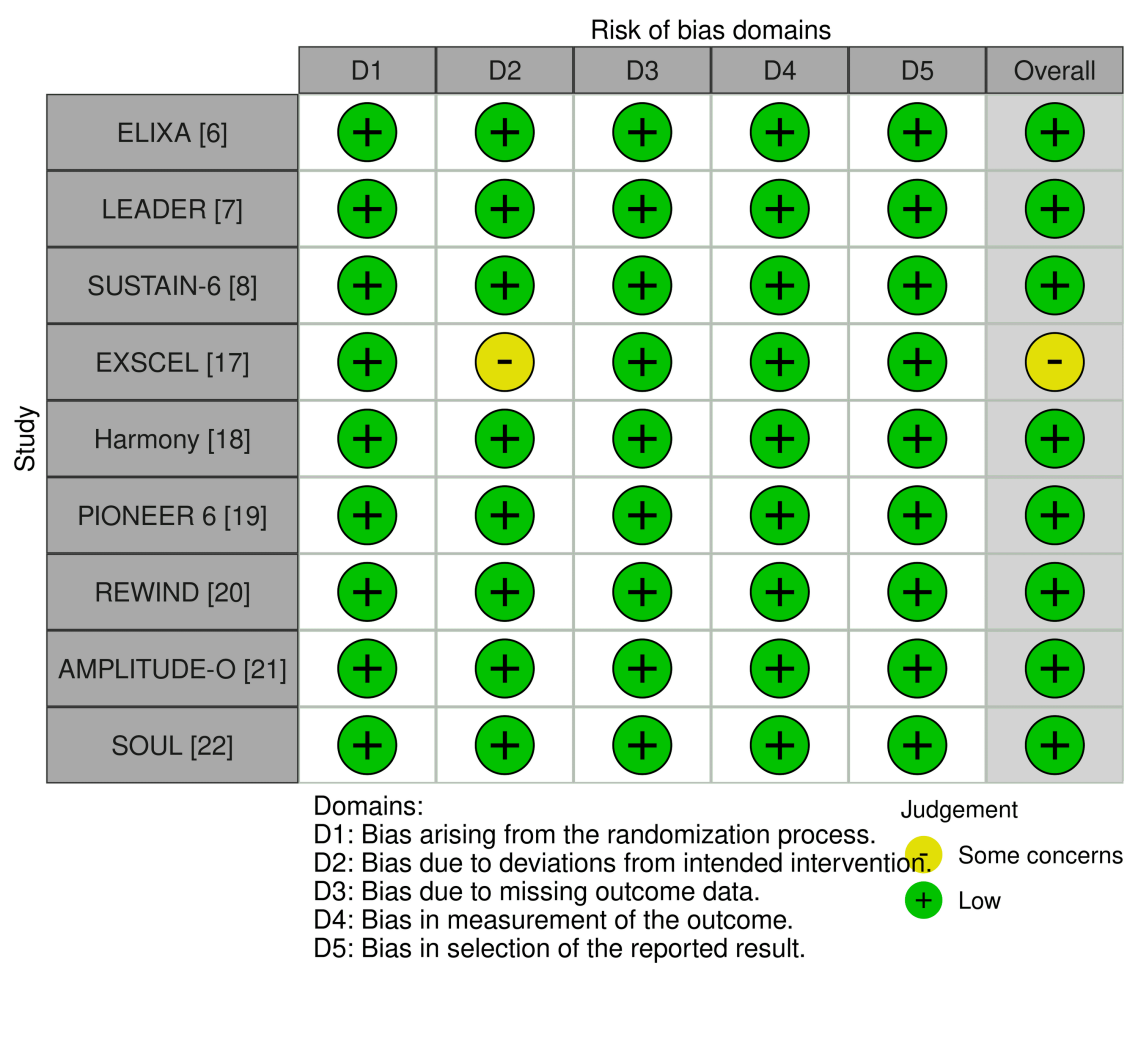
Risk-of-bias assessment (Cochrane RoB 2).

The risk-of-bias appraisal using RoB 2 is summarized in Figure 4. All included trials were large, multicenter, double-blind, placebo-controlled studies with central randomization and independent, blinded endpoint adjudication, and were judged at low risk of bias for randomization, outcome measurement, and selection of the reported result. EXSCEL raised some concerns in the domain of deviations from intended interventions, reflecting substantial premature discontinuation of study medication and open-label initiation of other glucose-lowering therapies during follow-up, factors likely to bias the efficacy estimate toward the null [17]. The overall body of evidence was assessed as being of high methodological quality.

Certainty of Evidence (GRADE)

The GRADE certainty of evidence by outcome is presented in Table 4. Certainty was rated high for the increase in gastrointestinal adverse events, which was large and consistent across all trials, and moderate for the principal cardiovascular efficacy and mortality outcomes, rated down predominantly for inconsistency in the magnitude of effect across agents and for imprecision in outcomes with fewer accrued events. The single-trial signal of diabetic retinopathy was rated as low certainty. A summary of findings table presenting anticipated absolute effects is provided as Table 5.

**Table 4:** GRADE certainty of evidence by outcome. GRADE certainty: ●●●● High; ●●●○ Moderate; ●●○○ Low; ●○○○ Very low. As all studies were randomized trials, each outcome began at high certainty. Participant totals are approximate and reflect the trials reporting each outcome. GRADE: Grading of Recommendations Assessment, Development, and Evaluation; GLP-1 RA: glucagon-like peptide-1 receptor agonist.

Outcome	Trials (participants)	Certainty (GRADE)	Effect	Reason for rating down
Major adverse cardiovascular events (3-point MACE)	9 (69,730)	Moderate (●●●○)	Favors GLP-1 RA	Inconsistency (effect size varies across agents, HR = 0.73-1.02)
Cardiovascular mortality	9 (69,730)	Moderate (●●●○)	Favors GLP-1 RA (modest)	Inconsistency (significant in some trials only)
All-cause mortality	9 (69,730)	Moderate (●●●○)	Favors GLP-1 RA (modest)	Inconsistency across trials
Nonfatal myocardial infarction	9 (69,730)	Moderate (●●●○)	Favors GLP-1 RA	Inconsistency (dominant driver in some trials only)
Nonfatal stroke	9 (69,730)	Moderate (●●●○)	Favors GLP-1 RA	Imprecision (fewer events; wide trial-level CIs)
Hospitalization for heart failure	≥6 (>50,000)	Moderate (●●●○)	No clear effect (neutral)	Imprecision
Gastrointestinal adverse events	9 (69,730)	High (●●●●)	Increased with GLP-1 RA	Not rated down (large, consistent effect)
Diabetic retinopathy complications	1 (3,297)	Low (●●○○)	Possible increase (semaglutide)	Indirectness (single agent/trial) and imprecision

**Table 5:** GLP-1 receptor agonists compared with placebo for the primary MACE outcome in adults with type 2 diabetes. Anticipated absolute effects are illustrative, derived by applying the pooled random-effects hazard ratio to a representative assumed control-group MACE risk of 120 per 1000 over the trials’ median follow-up; absolute benefit scales with baseline cardiovascular risk. GRADE: Grading of Recommendations Assessment, Development, and Evaluation; GLP-1: glucagon-like peptide-1; GLP-1 RA: glucagon-like peptide-1 receptor agonist; MACE: major adverse cardiovascular events; CI: confidence interval; HR: hazard ratio; NNT: number needed to treat; RCT: randomized controlled trial.

Outcome	Pooled relative effect (95% CI)	Anticipated absolute effects (per 1000)	Participants (trials)	Certainty (GRADE)
Major adverse cardiovascular events (3-point MACE)	HR = 0.86 (0.82–0.92)	Assumed risk with placebo: 120 per 1000. With GLP-1 RA: 104 per 1000 (98–110). 16 fewer per 1000 (10–22 fewer); NNT ≈ 62.	69,730 (9 RCTs)	Moderate (●●●○)

Discussion

This systematic review and meta-analysis of nine randomized, placebo-controlled CVOTs, encompassing nearly 70,000 participants with T2DM, demonstrates that GLP-1 RAs, considered as a class, reduce the risk of MACE by approximately 14% (pooled HR: 0.86, 95% CI: 0.82-0.92) while maintaining an acceptable, well-characterized safety profile. Eight of nine trials produced point estimates favoring the active agent, and five reached statistical superiority over placebo for the primary endpoint [7,18,20-22]. Yet the synthesis also reveals meaningful heterogeneity in the magnitude of benefit, in the endpoint that drives it, and in the populations in whom it was demonstrated.

Comparison With Previous Evidence

These findings are coherent with, and reinforced by, the broader evidence base. An early meta-analysis of the first four CVOTs estimated a MACE reduction of approximately 10% for the class [24], and a later, more comprehensive synthesis of eight trials reported a pooled MACE hazard ratio of 0.86 with significant reductions in cardiovascular mortality, all-cause mortality, stroke, myocardial infarction, and a composite kidney outcome [12]. Our pooled estimate of 0.86, now incorporating the 2025 SOUL trial, which was unavailable to those analyses, aligns almost exactly with this prior work, providing an internal validity check and indicating that the addition of contemporary oral semaglutide data does not alter the class-level conclusion. The convergence of independent pooled estimates on a 12-14% relative risk reduction lends confidence that the class effect is real and robust rather than an artefact of any single trial. The most instructive contrast within the dataset is between the trials that demonstrated benefit and the two that did not. ELIXA was entirely neutral, establishing only that lixisenatide did not increase risk after acute coronary syndrome [6]; its short-acting pharmacology, acute high-risk population, and short follow-up have each been advanced as explanations. EXSCEL failed to demonstrate superiority, although its point estimate was directionally favorable and its all-cause mortality result was nominally significant [17]; interpretation is complicated by high study-drug discontinuation and open-label drop-in of other agents, both of which dilute a true effect.

Mechanisms of Cardiovascular Benefit

The divergence in which the MACE component drives the composite is biologically informative and consistent with a multifactorial mechanism. In REWIND, and consistent with SUSTAIN-6, benefit was driven principally by stroke reduction, whereas in Harmony Outcomes and SOUL, the dominant contribution came from myocardial infarction [8,18,20,22]. This pattern suggests that the cardioprotective effect of GLP-1 RAs is mediated not by a single pathway but by a composite of anti-atherosclerotic, anti-inflammatory, blood-pressure-lowering, and weight-reducing actions whose relative expression varies with agent and population. Contemporary mechanistic reviews support this interpretation, describing GLP-1-receptor-dependent suppression of vascular inflammation, reduced leukocyte-endothelial adhesion, plaque stabilization, and improved endothelial function as central to the atheroprotective phenotype [4,5]. A further consideration is the apparent molecular divergence within the class: human GLP-1 analogues most clearly demonstrated benefit, whereas exendin-based agents were more variable, though AMPLITUDE-O complicates this dichotomy, since efpeglenatide, an exendin-based molecule, produced one of the largest reductions in the dataset (HR = 0.73) [21]. The most parsimonious reading is that structural homology is one contributor among several, with trial power, event accrual, and follow-up duration at least as important.

Clinical Implications

The clinical message of this synthesis is reflected in current guidelines. The most important recent extension of the evidence concerns populations beyond those captured here. The SELECT trial, correctly excluded because it enrolled patients with overweight or obesity and established cardiovascular disease but without diabetes, demonstrated a 20% reduction in MACE with subcutaneous semaglutide 2.4 mg (HR: 0.80, 95% CI: 0.72-0.90) [25], disentangling cardiovascular benefit from glucose lowering. The FLOW trial, which was excluded because its primary endpoint was a kidney composite, established that semaglutide reduces major kidney disease events in T2DM and chronic kidney disease [26]. These data are now embedded in practice: the American Diabetes Association Standards of Care 2025 [10] and the 2023 European Society of Cardiology guidelines [11] both recommend a GLP-1 RA with proven cardiovascular benefit for people with T2DM and established or high atherosclerotic risk, independent of baseline HbA1c or metformin use. It is equally important to situate these results alongside the SGLT2 inhibitors. EMPA-REG OUTCOME, DECLARE-TIMI 58, and the CANVAS Program established a class effect dominated by reductions in hospitalization for heart failure and renal progression [27-29]. Whereas GLP-1 RAs act predominantly on atherosclerotic events (myocardial infarction and stroke), SGLT2 inhibitors act most strongly on heart failure and renal endpoints, a domain in which GLP-1 RAs were largely neutral here [6,17]. This complementarity underlies contemporary recommendations favoring combination therapy in the highest cardiorenal risk, and prespecified analyses within AMPLITUDE-O and SOUL suggesting preserved GLP-1 RA benefit irrespective of background SGLT2 inhibitor use provide early randomized support [21,22].

Future Research Directions

Several questions remain incompletely resolved by the existing trial evidence and define priorities for future work. The retinopathy signal in SUSTAIN-6 has not been definitively explained, and whether it reflects a transient consequence of rapid glycemic improvement or a durable concern requires dedicated long-term ophthalmological follow-up [8]. The mortality benefit, clearest in LEADER, was not uniformly reproduced, and its dependence on baseline risk, follow-up duration, and event accrual deserves further study [7]. The relative contributions of weight loss, glycemic improvement, blood pressure reduction, and direct vascular effects remain only partially quantified, although mediation analyses and the diabetes-independent benefit in SELECT point to substantial weight-loss-independent and glucose-independent components [25]. Finally, dedicated head-to-head comparative-effectiveness trials, individual-patient-data meta-analyses, and prespecified subgroup analyses (by agent backbone, baseline risk, and background therapy) would be required before any definitive ranking of individual agents could be supported.

Limitations

This review has several limitations. First, although a quantitative meta-analysis of the primary MACE outcome has now been performed, the included trials differ substantially in agent pharmacology, dosing, enrolled population, baseline cardiovascular risk, primary endpoint definition (three-point versus four-point MACE), and follow-up duration; the pooled estimate should therefore be read as a class-level average rather than as a precise common effect, and the moderate heterogeneity (I² = 37%) and wide prediction interval (0.75-1.00) reflect this clinical diversity. Second, the quantitative synthesis was confined to the primary composite endpoint using published trial-level hazard ratios; individual-participant data were not available, secondary and safety outcomes were synthesized narratively because of inconsistent reporting, and prespecified subgroup and meta-regression analyses by agent class or baseline risk were not feasible with only nine trials. Third, assessment of publication bias by funnel plot and Egger's test is underpowered with fewer than 10 studies and should be interpreted with caution. Fourth, most trials were sponsored by the manufacturers of the respective agents, introducing potential funding-related bias, although independent blinded endpoint adjudication mitigates this concern. Fifth, the eligibility criteria deliberately excluded trials in populations without diabetes and trials with non-MACE primary endpoints; while justified, this restricts generalizability. Sixth, two records could not be retrieved owing to paywall restrictions; both appeared on the basis of their abstracts to be secondary analyses rather than independent CVOTs, and because all nine landmark trials were captured, their exclusion is unlikely to have affected the conclusions. Finally, the review was not prospectively registered, and trials published after the final search date (15 December 2025) are not represented.

## Conclusions

Among adults with T2DM, GLP-1 RAs as a class reduce the risk of MACE by approximately 14% (pooled HR: 0.86, 95% CI: 0.82-0.92), with several agents demonstrating significant superiority over placebo and an associated reduction in mortality, against a consistent and manageable safety profile dominated by gastrointestinal effects and a recognized but small excess of gallbladder and biliary disease. The benefit extends across the spectrum of cardiovascular risk, including primary-prevention-enriched populations, and is driven by reductions in atherosclerotic events (myocardial infarction and stroke) rather than heart failure. These findings, corroborated by class-level meta-analyses and extended by evidence in non-diabetic and chronic kidney disease populations, support the established role of GLP-1 RAs with proven cardiovascular benefit as a cornerstone of cardiovascular risk reduction in T2DM, ideally as part of an individualized strategy that incorporates complementary cardiorenal therapies. Between-agent and between-trial heterogeneity preclude a definitive ranking of individual agents.

## Appendices

**Table 6:** Full electronic search strategies.

Database/platform	Search string and limits
MEDLINE (PubMed)	(("glucagon-like peptide 1 receptor"[MeSH] OR "GLP-1 receptor agonist*"[tiab] OR lixisenatide OR liraglutide OR semaglutide OR exenatide OR albiglutide OR dulaglutide OR efpeglenatide) AND ("cardiovascular diseases"[MeSH] OR "major adverse cardiovascular event*"[tiab] OR MACE[tiab] OR "cardiovascular mortalit*"[tiab]) AND ("diabetes mellitus, type 2"[MeSH] OR "type 2 diabet*"[tiab]) AND (randomized controlled trial[pt] OR random*[tiab] OR placebo[tiab])). Filters: Humans.
Embase (Elsevier)	('glucagon like peptide 1 receptor agonist'/exp OR 'glp 1 receptor agonist':ti,ab OR lixisenatide:ti,ab OR liraglutide:ti,ab OR semaglutide:ti,ab OR exenatide:ti,ab OR albiglutide:ti,ab OR dulaglutide:ti,ab OR efpeglenatide:ti,ab) AND ('cardiovascular disease'/exp OR 'major adverse cardiovascular event':ti,ab OR mace:ti,ab) AND ('non insulin dependent diabetes mellitus'/exp OR 'type 2 diabet*':ti,ab) AND ('randomized controlled trial'/exp OR random*:ti,ab OR placebo:ti,ab). Limit: human.
Cochrane CENTRAL	("GLP-1 receptor agonist" OR lixisenatide OR liraglutide OR semaglutide OR exenatide OR albiglutide OR dulaglutide OR efpeglenatide) AND (MACE OR "cardiovascular outcome*") AND ("type 2 diabetes") in Title/Abstract/Keyword; Trials.
ClinicalTrials.gov	Condition: type 2 diabetes; Intervention: GLP-1 receptor agonist (and individual agent names); Study type: interventional; with results. Cross-checked against published reports.
